# Loss of c-Jun N-terminal kinase-interacting protein-1 does not affect axonal transport of the amyloid precursor protein or Aβ production

**DOI:** 10.1093/hmg/ddt313

**Published:** 2013-07-03

**Authors:** Alessio Vagnoni, Elizabeth B.C. Glennon, Michael S. Perkinton, Emma H. Gray, Wendy Noble, Christopher C.J. Miller

**Affiliations:** Departments of Neuroscience and Clinical Neurosciences, Institute of Psychiatry, King's College London, London, UK

## Abstract

Disruption to axonal transport is an early pathological feature in Alzheimer's disease. The amyloid precursor protein (APP) is a key axonal transport cargo in Alzheimer's disease since perturbation of its transport increases APP processing and production of amyloid-β peptide (Aβ) that is deposited in the brains of Alzheimer's disease patients. APP is transported anterogradely through axons on kinesin-1 motors. One favoured route for attachment of APP to kinesin-1 involves the scaffolding protein c-Jun N-terminal kinase-interacting protein-1 (JIP1), which has been shown to bind both APP and kinesin-1 light chain (KLC). However, direct experimental evidence to support a role of JIP1 in APP transport is lacking. Notably, the effect of loss of JIP1 on movement of APP through axons of living neurons, and the impact of such loss on APP processing and Aβ production has not been reported. To address these issues, we monitored how siRNA mediated loss of JIP1 influenced transport of enhanced green fluorescent protein (EGFP)-tagged APP through axons and production of endogenous Aβ in living neurons. Surprisingly, we found that knockdown of JIP1 did not affect either APP transport or Aβ production. These results have important implications for our understanding of APP trafficking in Alzheimer's disease.

## INTRODUCTION

The correct transport of protein and organelle cargoes through axons (axonal transport) is an essential requirement for proper functioning of neurons. Since most proteins are synthesized in neuronal cell bodies, axonal and synaptic cargoes need to be transported anterogradely through axons to their final destinations, whereas trophic factors and other cargoes are transported retrogradely from the synapse to the cell body. Indeed, perturbation of axonal transport is now known to contribute to the pathogenic process in some neurodegenerative diseases (see for reviews (1–4)).

Alzheimer's disease is the most common form of dementia, but the molecular mechanisms that cause disease are not fully understood. However, altered metabolism of amyloid precursor protein (APP) leading to changes in production of amyloid-β peptide (Aβ) is believed to be central to the disease process. Aβ is an approximately 40–42 amino acid peptide that is derived from APP by proteolytic processing and which is deposited in the brains of Alzheimer's disease patients within amyloid plaques. Generation of Aβ involves processing by β-site APP cleaving enzyme-1 (BACE1) and γ-secretase that cleave APP at the N- and C-termini, respectively, of the Aβ sequence. In addition, APP is processed by α- and γ-secretase but since α-secretase cleaves within the Aβ sequence, this route precludes Aβ production (see for review (5)). A large body of evidence suggests that at least some forms of Aβ have pathogenic properties (see for reviews (6,7)). As such, understanding the mechanisms that control APP processing and Aβ production has major relevance to Alzheimer's disease research.

One route for altering APP processing involves changes to its transport through axons. Thus, experimental disruption of anterograde axonal transport of APP increases production of Aβ (8–10). Moreover, since Aβ itself disrupts axonal transport (11–17), one suggestion is that damage to axonal transport promotes a toxic cycle of events. In this scenario, disruption to APP transport increases Aβ production which, in turn, induces more damage to axonal transport to further elevate Aβ production (8). As such, APP represents a key axonal transport cargo in Alzheimer's disease.

APP is transported anterogradely through axons on kinesin-1 molecular motors (8,18–20). Most functional kinesin-1 is a heterotetramer of two kinesin-1 motor proteins and two kinesin-1 light chains (KLCs). Kinesin-1 contains the motor domain, whereas the KLC is mainly involved in binding of cargoes (21). KLC is required for APP transport (8,22), but the mechanism by which APP attaches to KLC is not clear.

In one model, the intracellular C-terminus of APP binds directly to KLC to facilitate transport, but some studies have queried this finding (23,24). Recently, calsyntenin-1 (also known as alcadein-α) has been shown to be involved in APP transport (9,25). Calsyntenin-1 is a type-1 membrane-spanning protein that binds directly to KLC via its intracellular C-terminus, so as to mediate post-Golgi transport of a subset of vesicles through axons (26–30). In this model, APP does not directly attach to KLC or kinesin-1 motors but rather is loaded onto calsyntenin-1 containing vesicle carriers for transport (9,10,25). Finally, APP may attach to KLC via intermediary scaffolding proteins such as c-Jun N-terminal kinase-interacting protein-1 (JIP1) or PAT1 (27,31–36). JIP1 is the favoured scaffold since it binds to both APP and KLC in biochemical assays and a proportion of APP and JIP1 colocalize in axons (27,32,36–41). Moreover, axonal transport of APP involving JIP1 has been implicated in APP processing and Aβ production (27,41).

However, much of the data supporting a role of JIP1 in axonal transport and processing of APP involve analyses of fixed cells or cell lines overexpressing JIP1 and/or APP (27,32,35,36). Notably, the effect of loss of JIP1 on axonal transport of APP in living neurons and how such loss affects production of endogenous Aβ have not been reported. Clearly, a proper analysis of the role of JIP1 in APP transport requires dynamic studies of APP movement. Here, we address these issues by analyses of neurons in which JIP1 expression is downregulated with siRNAs.

## RESULTS

### siRNA loss of JIP1 does not influence Aβ production, APP processing at the α- or β-secretase sites or phosphorylation of APP at threonine-668

**Figure 1.**
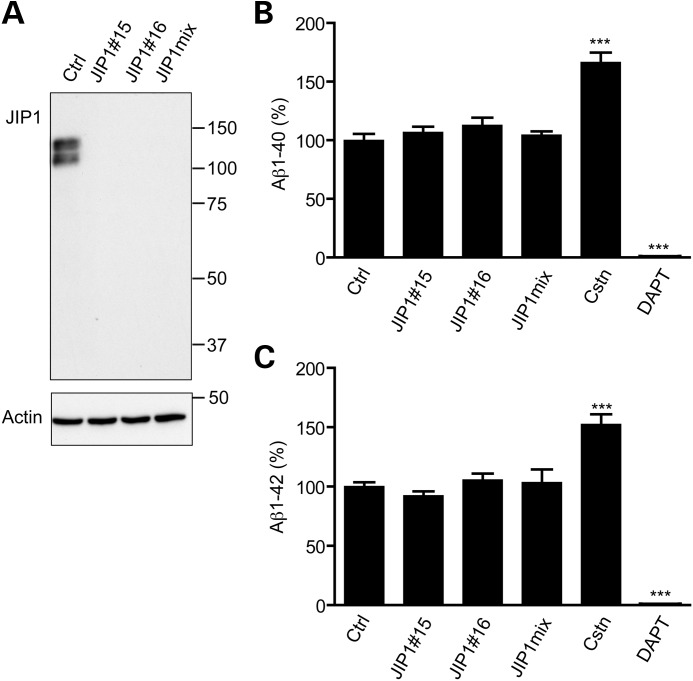
siRNA knockdown of JIP1 does not affect the production of Aβ. (**A**) siRNA knockdown of JIP1 in rat cortical neurons. Neurons were treated with control (Ctrl) siRNA or with two different JIP1 (JIP1#15 and JIP1#16) or a mixture of both JIP1 siRNAs (JIP1mix). Samples were then probed on immunoblots for JIP1 or actin as a loading control. Molecular mass markers are shown. (**B**) ELISAs showing relative Aβ(1–40) and Aβ(1–42) levels (expressed as a percentage of control values) in conditioned media from neurons treated with control siRNA, JIP1#15, JIP1#16, JIP1mix or calsyntenin-1 siRNAs. Also shown are neurons treated with DAPT. Statistical significance was determined by one-way ANOVA with a Bonferroni *post hoc* test. *N* = 6 error bars are SEM; ***P < 0.001.

We first tested the role of JIP1 in the production of endogenous Aβ in rat cortical neurons. To do so, we utilized siRNAs to knock down JIP1. In the neurons, JIP1 migrated as two major species which is consistent with previous reports and is probably the result of differential phosphorylation since JIP1 is a known phosphoprotein (42,43). Two different siRNAs and a mixture of both siRNAs all reduced JIP1 to undetectable levels (Fig. 1A). However, Aβ ELISAs revealed that this reduction in JIP1 did not affect secretion of either Aβ(1–40) or Aβ(1–42) into the media (Fig. 1B and C). For comparison and as controls, we also treated the neurons with siRNAs for calsyntenin-1 and with the γ-secretase inhibitor *N*-[*N*-(3,5-difluorophenacetyl)-1-alanyl]-*S*-phenylglycine-*t*-butyl ester (DAPT). Loss of calsyntenin-1 has been shown to disrupt axonal transport of APP to increase Aβ production, whereas DAPT inhibits γ-secretase cleavage of APP to decrease Aβ production (9,10,44). In agreement with these findings, siRNA knockdown of calsyntenin-1 using a pool of siRNAs previously shown to reduce calsyntenin-1 to levels <5% of that seen in control neurons (9) increased whereas DAPT decreased production of Aβ(1–40) and Aβ(1–42) (Fig. 1B and C).

**Figure 2.**
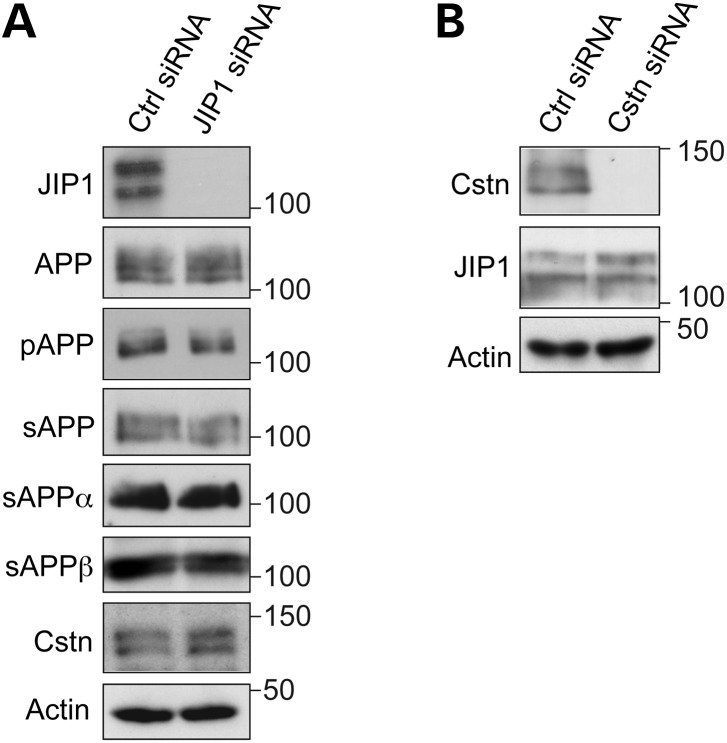
(**A**) siRNA knockdown of JIP1 does not affect APP processing or calsyntenin-1 levels in rat cortical neurons. Neurons were treated with control (Ctrl) or JIP1 mix siRNAs (JIP1) and the samples probed on immunoblots for full-length APP (APP), APP phosphorylated on threonine-668 (pAPP), total sAPP, sAPPα and sAPPβ in conditioned media, and calsyntenin-1 (Cstn); actin is shown as a loading control. No significant changes in the levels of any of these proteins or phosphorylation of APP on threonine-668 were detected between control or JIP1-siRNA treated neurons (Student's *t*-test *n* = 3). (**B**) siRNA knockdown of calsyntenin-1 does not affect JIP1 protein levels. Neurons were treated with control (Ctrl) or calsyntenin-1 (Cstn) siRNAs and the samples probed on immunoblots for JIP1 and actin as a loading control. No significant changes in the levels of JIP1 were detected between control or calsyntenin-1 siRNA-treated neurons (Student's *t*-test *n* = 3).

We next enquired whether siRNA knockdown of JIP1 influenced APP expression or its phosphorylation on threonine-668. JIP1 has been reported to preferentially bind to APP phosphorylated on threonine-668 (32). Since the two individual and mix of both JIP1 siRNAs all reduced JIP1 levels to the same extent (Fig. 1A), we utilized the siRNA mix for these experiments. siRNA loss of JIP1 did not alter total APP levels or the levels of APP phosphorylated on threonine-668 in the neurons (Fig. 2A).

We also enquired whether siRNA loss of JIP1 influenced APP processing at the α-secretase and BACE1 sites. Processing of APP by α-secretase and BACE1 generates N-terminal secreted APP fragments (sAPPα and sAPPβ), which can be detected in conditioned media from cells using antibodies that specifically recognize each sAPP fragment. Immunoblotting of conditioned media from control or JIP1 siRNA knockdown neurons with antibody 22C11 (that detects total sAPP) and antibodies that detect sAPPα and sAPPβ revealed that loss of JIP1 did not influence the levels of total sAPP, sAPPα or sAPPβ (Fig. 2A). These findings are consistent with the Aβ assays, which also suggest that JIP1 knockdown does not affect APP processing.

Finally, we enquired whether loss of JIP1 influenced calsyntenin-1 levels in the neurons since APP is transported through axons by kinesin-1 on calsyntenin-1 carriers and loss of calsyntenin-1 affects APP processing (9,10,25). However, loss of JIP1 had no discernible effect on calsyntenin-1 levels (Fig. 2A). Thus, loss of JIP1 in neurons does not influence endogenous APP levels, phosphorylation of endogenous APP at threonine-668, processing of endogenous APP at the α-secretase or BACE1 sites or production of endogenous Aβ.

Since loss of calsyntenin-1 affects APP processing, Aβ production and axonal transport of APP (9,10,25) (and see Fig. 1), we also monitored JIP1 levels in neurons treated with calsyntenin-1 siRNAs. However, we detected no difference in JIP1 levels between control and calsyntenin-1 siRNA knockdown neurons (Fig. 2B).

### siRNA loss of JIP1 does not noticeably influence axonal transport of APP

**Figure 3.**
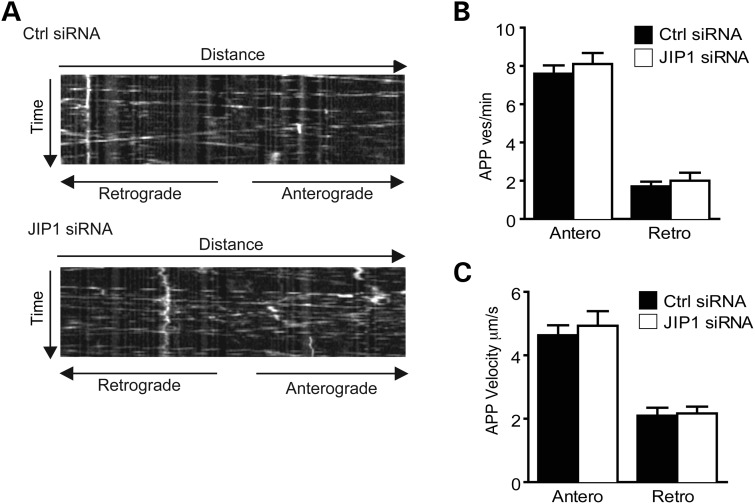
siRNA knockdown of JIP1 does not effect axonal transport of APP-EGFP. (**A**) Representative kymographs showing axonal transport of APP-EGFP in control and JIP1 siRNA-treated neurons. (**B**) and (**C**) show bar charts of APP-EGFP vesicle movement in control (Ctrl) and JIP1 siRNA treated neurons. (B) shows APP-EGFP flux rates (as APP vesicles/minute) and (C) shows APP-EGFP velocities in both anterograde and retrograde directions. Statistical significance was determined by Student's *t*-test. *n* = 26 control and 49 JIP1 siRNA treated cells. Error bars are SEM.

To examine the role of JIP1 in the axonal transport of APP, we quantified movement of enhanced green fluorescent protein (EGFP)-tagged APP (APP-EGFP) through axons of transfected living rat cortical neurons following siRNA knockdown of JIP1 using time-lapse microscopy. To avoid any possible artifacts produced by high levels of APP-EGFP expression, we chose for study, cells expressing low levels of APP-EGFP as judged by brightness of the EGFP signal. This approach is line with our previous studies (9,29,45). Kymographs were generated from the time-lapse movies, and these were used to calculate the velocities and flux rates of APP-EGFP movement in both anterograde and retrograde directions. Flux rates were determined by counting the numbers of APP-EGFP particles that crossed a defined line within the mid-section of axons as previously described by us and others (8,9,46). In control siRNA treated neurons, APP-EGFP moved through axons in a predominantly anterograde direction at fast speeds of between ∼1–9 μm/s and 1–5 μm/s in the anterograde and retrograde directions, respectively. These velocities and the bias towards anterograde movement are similar to those described previously for movement of fluorescent protein-tagged APP in neurons (8,9,18,19,46–48). siRNA knockdown of JIP1 did not significantly alter the velocities of APP-EGFP movement in either anterograde or retrograde directions, the bias towards anterograde movement, or the flux rates of anterograde or retrograde movements (Fig. 3) (and see Supplementary data). Thus, loss of JIP1 has no noticeable effect on axonal transport of APP through axons of living rat cortical neurons.

### The levels of JIP1 are not altered in Alzheimer's disease brains

**Figure 4.**
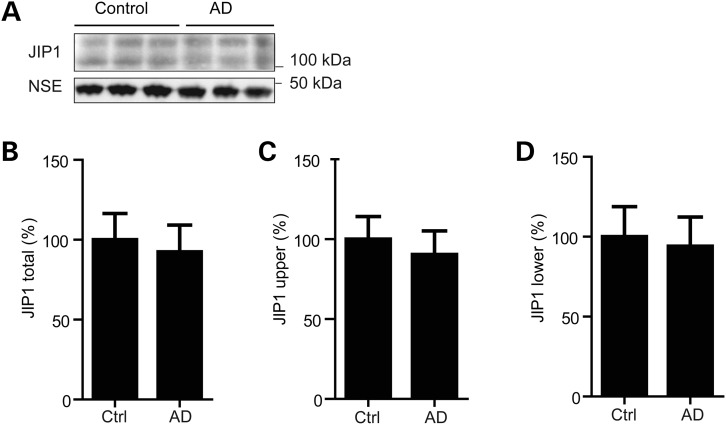
JIP1 levels are not altered in Alzheimer's disease brains. (**A**) Immunoblots of representative samples from control and Alzheimer's disease frontal cortex. Samples were probed for JIP1 and NSE. (**B**–**D**) Bar charts showing relative levels (%) of JIP1 in the two sample sets following normalization to NSE signals in each sample. Values shown are for total JIP1 (upper and lower species combined), upper JIP1 and lower JIP1 species. Data were tested for normality using a Kolmogorov–Smirnov normality test, and differences between groups determined by a Mann–Whitney test. *n* = 10 control and *n* = 19 Alzheimer's disease samples. Error bars are SEM.

To begin to determine whether JIP1 is altered in Alzheimer's disease, we monitored the levels of JIP1 protein in control and Braak stage V-VI Alzheimer's cortical brain samples (see Table 1, for details of patient samples). The age at death and post-mortem delay was not significantly different between the control and Alzheimer's disease samples. However, we found no differences in the levels of JIP1 between the two sample sets (Fig. 4).

**Table 1.** Data for human post-mortem samples showing age at death, post-mortem delay (PMD) and pathological diagnosis

Case	Age (years)	Sex	PMD	Pathological Diagnosis
A141/07	80	M	41	Alzheimer's disease Braak VI
A065/04	91	F	29	Alzheimer's disease Braak VI
A094/04	88	M	46	Alzheimer's disease Braak VI
A059/07	92	F	42	Alzheimer's disease Braak VI
A331/07	80	F	13	Alzheimer's disease Braak V
A191/07	69	F	16	Alzheimer's disease Braak VI
A210/05	84	F	<24	Alzheimer's disease Braak V
A167/05	81	F	<72	Alzheimer's disease Braak VI
A187/07	82	F	69	Alzheimer's disease Braak V
A168/05	84	F	36	Alzheimer's disease Braak V/VI
A247/05	90	F	23	Alzheimer's disease Braak V/VI
A350/09	98	F	3.5	Alzheimer's disease Braak V
A065/02	82	M	80	Alzheimer's disease Braak V/VI
A318/09	72	M	5	Alzheimer's disease Braak VI
A181/09	89	F	15	Alzheimer's disease Braak V
A240/06	97	F	12	Alzheimer's disease Braak V
A013/03	89	M	19	Alzheimer's disease Braak V
A122/04	86	M	26	Alzheimer's disease Braak V
A332/07	87	F	48	Alzheimer's disease Braak VI
A140/07	81	F	17	Control
A153/06	92	F	17	Control
A134/00	86	M	6	Control
A358/08	55	F	12	Control
A192/09	80	M	21	Control
A063/10	90	F	50	Control
A239/03	78	M	10	Control
A265/08	79	M	47	Control
A040/07	82	F	13	Control
A124/04	59	M	50	Control

## DISCUSSION

APP is transported anterogradely through axons by kinesin-1 motors (8,18–20), but the mechanisms that mediate attachment of APP to kinesin-1 are not properly understood. JIP1 is a ligand for KLC and via its PTB domain also binds to the C-terminus of APP (27,32,36–41). As such, JIP1 has been favoured as a scaffold to connect APP to KLC/kinesin-1 for anterograde axonal transport (27,32,35,36). Here, we directly tested the role of JIP1 in axonal transport of APP by quantifying APP-EGFP movement in JIP1 siRNA knockdown neurons. Despite highly efficient knockdown of JIP1, we detected no changes in APP movement in either anterograde or retrograde directions following loss of JIP1.

Disruption to axonal transport of APP influences its processing and production of Aβ (8–10). Others have shown that overexpression of JIP1 decreases Aβ production but these studies were conducted in non-neuronal or neuroblastoma cells overexpressing APP (27,41). Our analyses revealed no effect of loss of JIP1 on endogenous Aβ production in bona fide neurons, and these findings are consistent with the absence of an effect of JIP1 loss on axonal transport of APP in the same neurons. Of course, it is possible that JIP1 facilitates axonal transport of a proportion of APP on kinesin-1 motors but that in the JIP1 siRNA knockdown neurons, this effect is not noticeable due to switching of APP transport to other kinesin-1 carriers, such as those containing calsyntenin-1 so as to rescue any phenotype. Calsyntenin-1 has recently been shown to mediate transport of APP by kinesin-1 (9,10). Thus, while our results cannot formally eliminate any role of JIP1 in kinesin-1 transport of APP, the absence of any noticeable effect of loss of JIP1 on APP movement, APP processing or Aβ production together argue against a major role for JIP1 in axonal transport of APP at least in the cortical neurons utilized here.

In contrast to an absence of effect of JIP1, siRNA loss of calsyntenin-1 reduces axonal transport of APP to increase Aβ production (9,10) (and see Fig. 1). Calsyntenin-1 is a type-1 membrane-spanning protein that attaches via its intracellular C-terminus to KLC (26–28). Calsyntenin-1 also binds to the adaptor protein X11β (also known as X11-like and munc-18-interacting protein-2) (49). There is evidence that X11β functions as a coat protein to direct loading of cargoes into particular vesicle sub-types (50). X11β also binds to the C-terminus of APP (51,52) and like calsyntenin-1, modulating X11β expression affects APP processing and Aβ production (53–55). Thus, X11β may load APP onto calsyntenin-1 containing vesicles for post Golgi transport by kinesin-1 through axons and disruption to this transport either via loss of calsyntenin-1 or X11β may promote amyloidogenic processing of APP. JIP1 has been reported to be mislocalised in Alzheimer's disease (56) but we detected no changes in the total levels of JIP1 in Alzheimer's disease brains. This contrasts with calsyntenin-1 where both decreased protein levels and altered processing have been reported (9,57,58). Thus, defective axonal transport of APP in Alzheimer's disease may be more closely linked to defects in calsyntenin-1 rather than JIP1 mediated cargo transport.

## MATERIALS AND METHODS

### Plasmids and siRNAs

APP-EGFP was as described (9,59). Verified non-targeting control and rat JIP1 siRNAs (#15 5′-CUGCUAUGCAAAAGAUCGC-3′, #16 5′- CCAUGUUUUGGAGAUUACA-3′) were purchased from Dharmacon (Accell range). Calsyntenin-1 pool siRNAs were as described (9) and also obtained from Dharmacon.

### Antibodies

Rabbit APP C-terminal and calsyntenin-1 antibodies were as described (9,29,60,61). Mouse monoclonal antibodies to beta actin (clone AC-15) were from Abcam (Cambridge, UK) and a JIP-1 mouse monoclonal antibody (clone B-7) was from Santa Cruz Biotechnology (Santa Cruz, USA). Anti-APP mouse clone 22C11 was from Merck Millipore (Billerica, USA); anti-sAPPα clone 2B3 and anti-sAPPβ rabbit polyclonal antibodies were from IBL International (Hamburg, Germany), anti-phospho-threonine-668 APP rabbit polyclonal antibody was from Cell Signaling Technology (Danvers, USA). Anti NSE antibody was from Dako (Cambridge).

### Cell culture and transfection

Cortical neurons were obtained from embryonic day 18 rat embryos and cultured in Neurobasal medium containing B27 supplement, 100 IU/ml penicillin, 100 μg/ml streptomycin and 2 mm L-glutamine (Invitrogen). For siRNA studies, neurons at DIV4 were treated with 1 μm of each siRNA (control, JIP1 or calsyntenin-1) for 96 h and then analysed at DIV8. For transfection studies, neurons were cultured on poly-l-lysine-coated glass cover slips in 12-well plates. Neurons were transfected on a magnetic plate for 30 min using 1 μg plasmid DNA and 2 μl magnetic nanoparticles (NeuroMag, OZ Biosciences) as described by the manufacturer. Neurons were transfected on DIV7 and analysed on DIV8.

### Measurement of Aβ production

To determine how loss of JIP1 affects production of endogenous Aβ(1–40) and Aβ(1–42), conditioned medium from rat cortical neurons at DIV4 cultured in 24-well plates (2.5 × 10^5^ cells per well) was removed and replaced with 400 μl fresh Neurobasal/B27 medium containing 1 μm control, JIP1 or calsyntenin-1 siRNAs. The neurons were then returned to the incubator for a further 96 h and the conditioned media then assayed for Aβ(1–40) and Aβ(1–42). Some neurons were also treated with 2 μM of the γ-secretase inhibitor DAPT. For Aβ assays, the conditioned media was removed and centrifuged at 100000 × g for 30 min at 4°C to remove any insoluble material. Levels of Aβ(1–40) and Aβ(1–42) were then determined using quantitative mouse/rat Aβ ELISAs from Invitrogen (Aβ(1–40) ELISA KMB3481; Aβ(1–42) ELISA KMB3441) according to the manufacturer's instructions. Data were analysed using one-way ANOVA tests with Bonferroni *post hoc* test.

### SDS–PAGE and immunoblotting

Cells were harvested for sodium dodecyl sulphate–polyacrylamide gel electrophoresis (SDS–PAGE) by washing with phosphate buffered saline pre-warmed at 37°C and scraping into SDS–PAGE sample buffer and immediately heating to 100°C. Total sAPP, sAPPα and sAPPβ levels in conditioned media were likewise prepared for SDS–PAGE by addition of sample buffer at heating to 100°C.

Human brain samples were prepared as 20% homogenates and equal protein concentrations then prepared for SDS–PAGE by addition of SDS–PAGE sample buffer and heating to 100°C. Samples were separated on either 8 or 10% (w/v) acrylamide gels; separated proteins were then transferred to Protran nitrocellulose membranes (Schleicher & Schuell) using a Transblot system (BioRad) and following blocking, probed with primary antibodies. Following washing, the blots were further probed with horseradish peroxidase-conjugated goat anti-mouse or anti-rabbit Igs and developed using an enhanced chemiluminescence system (GE Healthcare).

### Human tissues

All human tissue collection and processing were carried out under the regulations and licensing of the Human Tissue Authority and in accordance with the Human Tissue Act, 2004.

### Time-lapse microscopy

Live microscopy of APP-EGFP was performed with an Axiovert S100 microscope (Zeiss) equipped with a Lambda LS Xenon-Arc light source (Sutter Instrument Company, Novato, USA), GFP filter set (Chroma Technology Corp., Rockingham, USA), 100× Plan-Apochromat 1.4 N.A. objective (Zeiss), Lambda 10–3 filter wheel (Sutter Instrument Co.) and a Photometrics Cascade-II 512B High Speed EMCCD camera (Photometrics, Tuscon, USA). Twenty-four hour post-transfection, the cells were transferred to a Ludin imaging chamber (Life Imaging Systems, Basel, Switzerland) mounted on the stage of the microscope. Neurons were maintained in a HEPES-buffered extracellular neuronal solution (composition in mm: naCl, 140; KCl, 5; NaHCO_3_, 5; MgCl_2_.6H_2_0, 1; CaCl_2_, 1.2; Na_2_HPO_4_, 1.2; Glucose, 10; Hepes, 20; pH 7.4) at 37°C using an objective heater (IntraCell, Royston, UK) and ‘The Box’ Microscope Temperature Control System (Life Imaging Systems). Vesicle movements were recorded for 2 min with a 1 s time-lapse interval using MetaMorph software (Molecular Devices). Image analysis was performed with ImageJ. The flux rates for APP-EGFP transport in both anterograde and retrograde directions were determined essentially as described (8,9,46). This involved quantifying the numbers of APP-EGFP particles that crossed a defined line in mid-axons per minute. APP-EGFP velocities were determined from kymographs, essentially as described for other cargoes (62). Statistical significance was determined by Student's *t*-test.

## SUPPLEMENTARY MATERIAL

Supplementary Material is available at *HMG* online.

## FUNDING

This work was supported by grants from Alzheimer's Research UK, MRC and the Wellcome Trust. Funding to pay the Open Access publication charges for this article was provided by the Wellcome Trust.

## Supplementary Material

Supplementary Data
