# Supplementary material for: Loss of c-Jun N-terminal kinase-interacting protein-1 does not affect axonal transport of the amyloid precursor protein or Aβ production
Source: Hum Mol Genet. 2013 Jul 3;22(22):4646–52. doi: 10.1093/hmg/ddt313 (PMC3889811; doi:10.1093/hmg/ddt313)
Supplement: Supplementary Data [file supp_22_22_4646__index.html]

Loss of c-Jun N-terminal kinase-interacting protein-1 does not affect axonal transport of the amyloid precursor protein or Aβ production — Loss of c-Jun N-terminal kinase-interacting protein-1 does not affect axonal transport of the amyloid precursor protein or Aβ production — Supplementary Data 

# Loss of c-Jun N-terminal kinase-interacting protein-1 does not affect axonal transport of the amyloid precursor protein or Aβ production

## 

Supplementary Data

**Files in this Data Supplement:**

- Supplementary Data - Doc file
- Supplementary Movie 1 - mov file
- Supplementary Movie 2 - mov file
