# Supplementary material for: Loss of c-Jun N-terminal kinase-interacting protein-1 does not affect axonal transport of the amyloid precursor protein or Aβ production
Source: Hum Mol Genet. 2013 Jul 3;22(22):4646–52. doi: 10.1093/hmg/ddt313 (PMC3889811; doi:10.1093/hmg/ddt313)
Supplement: Supplementary Data [file supp_ddt313_ddt313supp_data.doc]

**Supplementary data**

Supplementary movies 1 and 2. Quicktime movies of APP-EGFP movement in axons from neurons treated with control (supplementary movie 1) or JIP1 siRNAs (supplementary movie 2). Movement from left to right represents anterograde movement. Movie length 2 minutes; time-lapse period 1 second.
